# Renal necrotic epithelioid angiomyolipoma: A case report

**DOI:** 10.1016/j.eucr.2026.103454

**Published:** 2026-04-22

**Authors:** Gede Wirya Kusuma Duarsa, Pande Made Wisnu Tirtayasa, I Wayan Juli Sumadi, Kadek Budi Santosa, I Wayan Yudiana, Ida Bagus Putra Pramana, Nyoman Gede Prayudi

**Affiliations:** a Department of Urology, Faculty of Medicine Universitas Udayana, Department of Uro-Nephrology, Ngoerah Hospital, Bali, Indonesia; b Department of Urology, Faculty of Medicine Universitas Udayana, Universitas Udayana Hospital, Bali, Indonesia; c Department of Anatomical Pathology, Faculty of Medicine Universitas Udayana, Ngoerah Hospital, Bali, Indonesia

**Keywords:** Renal angiomyolipoma, Epithelioid variant, Renal neoplasm

## Abstract

Epithelioid angiomyolipoma is a rare variant of angiomyolipoma, with necrosis being one of its unfavourable prognostic indicators. We report a 39-year-old woman with necrotic epithelioid angiomyolipoma of the left kidney. Left Radical nephrectomy was performed and thorough follow-up was undertaken even though no sign of recurrences or distant metastasis was found.

## 1 Introduction

Angiomyolipoma (AML) is the most common form of mesenchymal neoplasm of the kidney. AML is generally a benign condition that normally comprises three components: mature adipose tissue, dysmorphic blood vessels, and smooth muscle fibers.^1^ AML is classified into two primary subtypes: the more common classical subtype and the rare, more aggressive epithelioid angiomyolipoma (EAML). Necrotic EAML is a rare, potentially malignant variant of renal AML distinguished by a high prevalence of epithelioid cells, marked cytologic atypia, and necrotic areas.^2^ Herein, we report a case of necrotic EAML of the kidney.

## 2 Case presentation

**Fig. 1:**
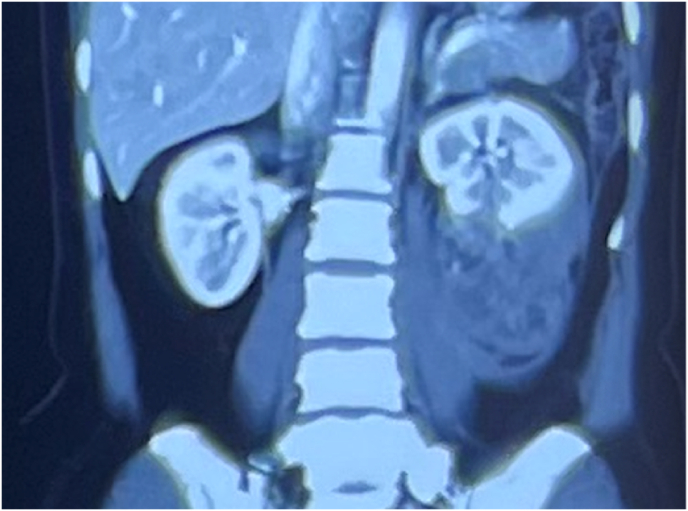
Coronal abdominal CT scan image with contrast. Sporadic patches of necrosis and calcification were observed.

**Fig. 2:**
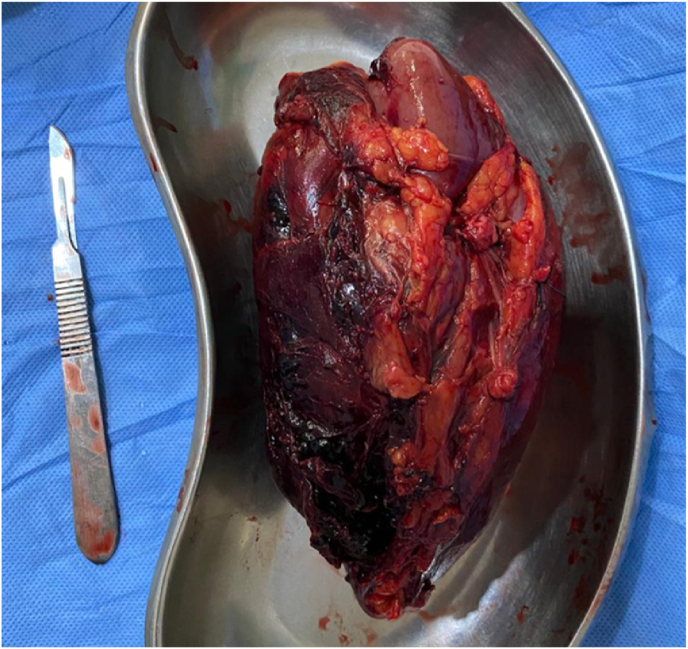
Gross appearance of the neoplasm. Mass affixed to the inferior pole of the kidney, exhibiting regions of necrosis and haemorrhage, infiltrating the renal sinus.

**Fig. 3:**
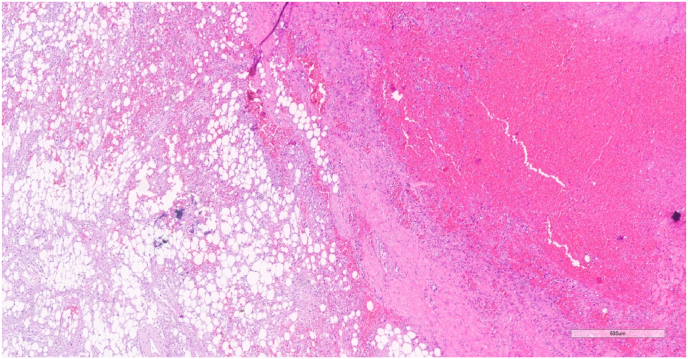
Microscopic appearance of the specimen (H&E x 40). A composite of smooth muscle, adipose tissue, and blood vessels was noted that exhibits a characteristic of epithelioid morphology. Sign of haemorrhage was also observed.

A 39-year-old woman complained of intermittent, moderate pain in the left lumbar area without haematuria. The past medical history was unremarkable, and she denied having a family or personal history of genitourinary illness. Vital signs and physical examination were within normal limits without detectable mass or tenderness in the lumbar area. The urinalysis revealed the absence of blood, leukocytes, and protein. Abdominal ultrasound identified a significant hypoechoic mass in the lower pole of the left kidney. Computed tomography (CT) with contrast of the abdomen and chest revealed a mass of heterogeneous density measuring 8 × 7 cm, located at the lower pole of the left kidney. Occasional scattered necrosis and calcification were noted, and the lesion demonstrated moderate heterogeneous enhancement during contrast imaging (Fig. 1). Initially, the mass was radiographically identified as renal cell carcinoma. The examination revealed infiltration of the peri-renal fat without nodules detected in the liver or lungs. Radical left nephrectomy was performed, and gross pathology examination revealed an 8 × 7 × 6 cm mass attached to the lower pole of the kidney with areas of necrosis and haemorrhage infiltrating the renal sinus. The renal capsule remained uninvaded, and no tumour thrombus was observed in the renal vein (Fig. 2). Histologic examination of the mass demonstrated a combination of smooth muscle, adipose tissue and various thicknesses of blood vessels. The smooth muscle cells had prominent nuclei and a round to oval shape that was consistent with epithelioid morphology (Fig. 3).

## 3 Discussion

EAML is an uncommon variant of AML that demonstrates more aggressive characteristics than the classical subtype.^2^ Renal AML may arise sporadically or in conjunction with other conditions, predominantly tuberous sclerosis. On CT, AMLs are frequently well-defined and generally measure −10 Hounsfield units or lower. Intralesional necrosis on CT is uncommon but characteristic of epithelioid variants.^3^ Necrosis was observable on imaging in the present case. AML typically comprises three components: mature adipose tissue, dysmorphic blood vessels, and smooth muscle fibres. EAML typically comprises primarily sheets of epithelioid cells exhibiting significant cytoplasmic pleomorphism and atypia, as present in our case.

There are no histological criteria for malignant EAML, except for distant metastases, which are recognised as definitive indicators of malignancy. Numerous recent studies investigated histological parameters indicative of malignant behaviour. A previous study compared histological criteria between progressor and non-progressor patients. A significant proportion of epithelioid cells, notable atypia, and increased mass diameter were indicative of tumour progression. The five unfavourable prognostic indicators of EAML were tuberous sclerosis complex or concurrent AML, necrosis, tumour size exceeding 7 cm, extra-renal extension and/or renal vein involvement, and carcinoma-like growth pattern.^2^ Furthermore, a Ki67 index surpassing 10%, along with P53 overexpression and mutations in EAML, may signify the tumour's malignant behaviour.^4^

The management of EAML remains debatable due to its rarity and unpredictable clinical behaviour. Surgical resection is the primary treatment modality, with partial nephrectomy favoured for small, localised tumours to preserve renal function.^5^ Nonetheless, diligent surveillance is essential owing to the potential for metastasis and recurrence. Even though our patient possessed unfavourable prognostic indicators, she exhibited no signs of recurrence or distant metastasis at the 24-month follow-up.

## 4 Conclusions

EAML are uncommon and potentially malignant, exhibiting a propensity for recurrence and frequent metastasis. Consequently, vigilant surveillance should be conducted following definitive treatment.

## CRediT authorship contribution statement

**Gede Wirya Kusuma Duarsa:** Writing – review & editing, Writing – original draft, Visualization, Validation, Supervision, Methodology, Investigation, Funding acquisition, Formal analysis, Data curation, Conceptualization. **Pande Made Wisnu Tirtayasa:** Writing – review & editing, Writing – original draft, Visualization, Validation, Software, Resources, Methodology, Investigation, Funding acquisition, Formal analysis, Data curation, Conceptualization. **I Wayan Juli Sumadi:** Visualization, Validation, Supervision, Software, Investigation. **Kadek Budi Santosa:** Visualization, Validation, Supervision, Project administration. **I Wayan Yudiana:** Visualization, Validation, Supervision. **Ida Bagus Putra Pramana:** Visualization, Validation, Supervision. **Nyoman Gede Prayudi:** Validation, Supervision.
